# Supplementary material for: Navigated ultrasound bronchoscopy with integrated positron emission tomography—A human feasibility study
Source: PLoS One. 2024 Aug 30;19(8):e0305785. doi: 10.1371/journal.pone.0305785 (PMC11364294; doi:10.1371/journal.pone.0305785)
Supplement: S1 File — (PDF) [file pone.0305785.s001.pdf]

Measurements of CT to ultrasound accuracies

	Sites	All measurements (mm)
Patient 1	Aorta top	6,68
	Aorta right	7,74
	Aorta left	7,20
	Azygos cente	15,32
	11R 1	10,94
	11R 2	13,25
	11R 3	8,25
	11R 4	4,85
	11R 5	3,88
Patient 2	2R 1	7,86
	2R 2	8,31
	2R 3	10,49
	4R 1	6,74
	4R 2	7,19
	Subclavian a	8,18
Patient 3	Aorta top	4,24
	Aorta bottom	2,39
	Azygos cente	4,96
	2R	11,96
	10R 1	3,14
	10R 2	6,83
	10R 3	3,45
	10R 4	3,08
	10R 5	3,47
